# Measuring the course of anxiety in women giving birth by caesarean section: a prospective study

**DOI:** 10.1186/s12884-016-0906-z

**Published:** 2016-05-18

**Authors:** Philip Hepp, Carsten Hagenbeck, Bettina Burghardt, Bernadette Jaeger, Oliver T. Wolf, Tanja Fehm, Nora K. Schaal, The MAGIC Group

**Affiliations:** Clinic for Gynecology and Obstetrics, Heinrich-Heine-University, Düsseldorf, Germany; Department of Cognitive Psychology, Institute of Cognitive Neuroscience, Faculty of Psychology, Ruhr-University Bochum, Bochum, Germany; Department of Experimental Psychology, Heinrich-Heine-University, Düsseldorf, Germany; Clinic for Gynecology and Obstetrics, HELIOS Universitätsklinikum Wuppertal, University Witten/Herdecke, Vogelsangstr. 109, 42109 Wuppertal, Germany

**Keywords:** Caesarean section, Anxiety, STAI questionnaire, Visual analog scale, Saliva cortisol, Saliva

## Abstract

**Background:**

Women undergoing elective caesarean section experience anxiety. However, course, extent and duration of anxiety have not been investigated yet. This study aimed to explore anxiety levels during the course of the day of surgery by employing and comparing subjective as well as objective measures. By examining their correlation it is intended to give methodological support for interventional studies.

**Methods:**

This is a monocentric, prospectively planned study in which 47 women with an indication for primary caesarean section took part. Anxiety levels were evaluated using the State-Trait Anxiety Inventory (STAI-trait and STAI-state), the visual analogue scale for anxiety (VASA) as well as saliva cortisol at three time points on the day of the caesarean section (at admission, at skin closure and 2 h post surgery).

**Results:**

Peak anxiety levels for the STAI-state and VASA were highest at admission and showed significant decreases to skin closure (*p* < .001). The subjective measures correlated significantly at all time points (*p*-values < .001). For cortisol levels the peak level of anxiety was shown at skin closure with a significant increase from admission to skin closure and a significant decrease from skin closure to 2 h post operation (*p*-values < .001). Additionally women with STAI-trait scores above the median showed significantly higher levels at the peaks of anxiety.

**Conclusion:**

The study reveals the course of anxiety on the day of the caesarean section. A strong correlation of STAI-state and VASA was demonstrated. Cortisol showed a different course, which fits into its known biological kinetics. Taking into account all measures, anxiety seems to be most bothersome before surgery until skin closure. In a differentiated approach using STAI-trait scores as a discriminator we showed that the group with STAI-trait levels above the median is particularly prone to develop anxiety in the setting of the caesarean section and might therefore mostly be in need of an intervention against anxiety.

## Background

In 2014, 31.8 % of all births in Germany were by caesarean section (CS) [1]. Throughout the world, it is one of the most common surgeries in obstetrics and gynaecology. In contrast to most other surgeries, CS is usually performed in healthy, pregnant women. With regard to a minimized exposition of the newborn to anesthetics and the parents’ desire to witness the birth, the majority of women undergo the operation with regional anesthesia without anxiolytic or sedating medication.

Even though data about consequences for the mother and the newborn in terms of physical detriments are vastly available and part of every informed consent, only few studies have elucidated the impact of CS on psychological parameters [2]. Mostly, women see CS as a routine procedure and may be neglecting the potential psychological side effects and the discomfort it may cause [3]. Sparse measurements already showed that preoperative anxiety is associated with reduced satisfaction and worse recovery from CS [4].

A study by Wyatt et al. reported high preoperative anxiety levels in women before an elective CS under regional anaesthesia [5]. To the best of our knowledge, no data exist so far reporting the course of anxiety from before CS until thereafter taking into account subjective (i.e. questionnaires) as well as objective (i.e. anxiety related hormones) parameters. Nevertheless, tools for the quantification of anxiety are at hand, one of the most frequently used being the “State-Trait Anxiety Inventory” (STAI), an introspective inventory comprising 40 self-report items pertaining to anxiety [6]. It distinguishes between two questionnaires with 20 items each, one measuring anxiety perceived in the current situation (STAI-state) and the other evaluating a general tendency towards anxiety (STAI-trait). The STAI-trait questionnaire is not included that often in clinical studies even though it has been shown that it can potentially be used to identify patients who are prone to high levels of anxiety in surgical settings [7] as well as who could benefit most from additional interventions reducing anxiety in medical settings [8]. On the other hand, the STAI-state questionnaire has been used frequently in order to evaluate anxiety levels in clinical settings [9–12]. However, in a surgery setting like CS the repeated evaluation of the 20 items test (STAI-state) is not practicable. Therefore effort has been made to establish a more easily repeatable tool. There is evidence that a simple visual analogue scale depicting anxiety (VASA) predicts STAI-state results reliably [13, 14]. Nevertheless, a correlation of VASA and STAI-state has not been shown in the context of CS.

Looking at objective measures to evaluate anxiety, cortisol levels are often referred to [15]. Its relation to the activation of the hypothalamus-pituitary-adrenal axis (HPAA) is well understood [16]. Although it can be obtained from all kinds of specimens like blood, urine or hair, the sampling in saliva is the most common way inanxiety research as it reflects the activation of the HPAA within the last half hour before the specimen was obtained [17, 18]. Data about the correlation of cortisol and subjective measurements of anxiety are contradictory [19].

Therefore, we aimed to quantify anxiety in women having a CS by repeated measurements at three time points in order to assess the potential psychological impact of CS in regard to anxiety. Furthermore we intended to clarify the correlation of different subjective and objective measurements of anxiety in the context of CS. Finally, we explored whether STAI-trait scores could serve as a predictor to identify those patients who could benefit the most from an intervention in order to reduce anxiety or could be in need of postoperative psychological treatment.

## Methods

### Study design

The analysis is based on a monocentric, prospectively planned study at the Clinic for Gynecology and Obstetrics at the University Hospital in Duesseldorf, Germany. Patients scheduled for a planned, primary caesarean section with sufficient command of German language to complete the questionnaires were invited to participate. Data were collected from March through June 2015. In this period 47 women were enrolled in the study. The indications for the CS were previous caesarean sections (*N* = 23), breech presentation (*N* = 13), upon patient’s request (*N* = 6) and preexisting conditions of the mother (e.g. fracture of the pelvis, fibroids; *N* = 5). Two women did not undergo cesarean section because of spontaneous cephalic version from breech presentation and were therefore excluded from further analysis.

**Fig. 1:**
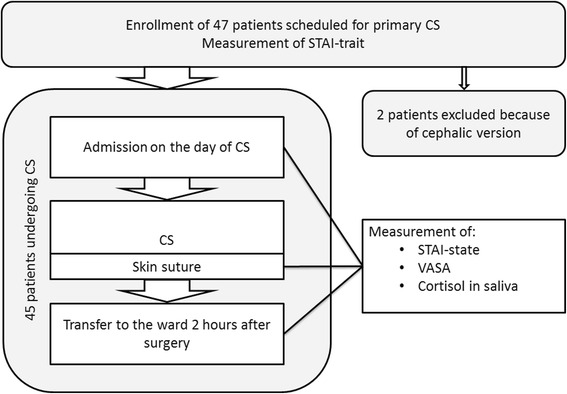
Flow chart of the study

Figure 1 shows a flow chart of the study. During preoperative visitation one to two weeks prior to scheduled surgery, participants were asked to fill in the STAI-trait questionnaire [6].

Each of the following measurements comprised a STAI-state questionnaire (measuring anxiety levels at the moment of evaluation), a visual analog scale depicting anxiety (VASA) and a saliva specimen for cortisol analysis. For the saliva sample patients were asked to thoroughly insalivate a cotton swab.

The first measurement took place at admission on the day of the surgery. The second measurement was taken during skin closure while the child already had left the surgery theater. Two hours after completion of surgery a third and last measurement was conducted before the patient was transferred to the postnatal ward according to the standard procedure in Germany.

### Ethics, consent and permissions

Written informed consent was obtained from all included patients. The study was approved by the ethics committee of the Heinrich-Heine-University Duesseldorf, Germany (protocol number 3625).

### Statistics

Sample size was calculated using the software G*power [20] with an estimated medium effect size, a power of 0.9 and with an alpha error probability of 0.05.

The analysis was performed using IBM SPSS Statistics® Ver. 22. The scores of the STAI-trait as well as STAI-state at the three time points were compared to the standard normative data of the German Version of the STAI [21] using one sample t-tests. Repeated measures ANOVAs were conducted independently for the three dependent variables (STAI-state, VASA and cortisol) with *time point* (admission vs. Skin closure vs. 2 h post caesarean) as the within-subject variable. Additionally planned post-hoc paired-sample t-tests were applied in order to disentangle significant main effects. Furthermore for a more detailed analysis the between-subject variable *STAI-trait group* (splitting the sample above/below the median = 36 of the STAI-trait score of our sample which is comparable to the mean value of 36.85 of the normative data [21]) was added to the ANOVAs. Here, planned post-hoc independent sample t-tests were conducted to disentangle the interactions.

In order to disclose possible correlations between the dependent variables Pearson product–moment bivariate correlations were calculated.

Greenhouse-Geisser corrections are reported when sphericity was violated. Bonferroni corrections were applied adequately throughout the analysis in order to avoid alpha I error associated with multiple testing.

Additionally, we ran the whole analysis using non-parametric tests (e.g. Friedman’s ANOVAs and Wilcoxon tests) as normality was not met for all of the dependent variables. But as the results were comparable to the parametric tests, we only report the results of the parametric tests, as they are more robust.

## Results

Data are available from 45 women with an average age of 35.5 years (SD = 5.1). For the analysis including the factor *STAI-trait group*, 40 women are included as data were missing from five participants.

### Comparison with normative data

No significant difference (*t*(39) = −.827, *p* = .414) was observed between STAI-trait scores of our sample (M = 35.9, SD = 8.5) and the standard normative value for females (M = 36.85) [21]. The STAI-state at admission (M = 47.35, SD = 10.83) was significantly above the standard value of 38.08 [21], (*t*(42) = 5.61, *p* < .001). At skin closure (M = 33.96, SD = 7.43) and 2 h post surgery (M = 31.11, SD = 7.74) the scores of the present sample were below the standard value (skin closure: *t*(44) = −3.72, *p* = .001; 2 h post surgery: *t*(43) = −5.97, *p* < .001).

### Analysis of variance

Three independent repeated measures ANOVAs with *time point* as the within-subject factor and STAI-state, VASA and cortisol respectively as the dependent variables were conducted and showed a significant main effect of *time point* on all three variables (STAI-state: *F*(2, 82) = 55.39, *p* < .001, *ηp*^*2*^ = .575; VASA: *F*(1.60, 55.81) = 42.68, *p* < .001, *ηp*^*2*^ = .549; Cortisol: *F*(1.40, 35.06) = 34.74, *p* < .001, *ηp*^*2*^ = .582). Planned post-hoc paired-sample t-tests for STAI-state and VASA showed that subjective anxiety levels were highest at admission before the caesarean (STAI-state 47.13 [SD = 9.57]; VASA 5.01 cm [SD = 3.14]) and that anxiety levels significantly decreased (*p* < .001 for both measures) from admission to skin suture (STAI-state 33.49 [SD = 7.49]; VASA 1.56 cm [SD = 2.06]). After the caesarean section no further significant reduction of the subjective measures could be revealed (*p* > .14) with STAI-state being 30.54 [SD = 7.05] and VASA 1.21 cm [SD = 1.90] 2 h post caesarean. Cortisol levels showed their peak at skin suture (26.67 μg/L [SD = 10.88]) with a significant increase from admission (12.62 μg/L [SD = 3.85]) to skin suture (*p* < .001) and a significant decrease from skin suture to 2 h post caesarean section (12.97 μg/L [SD = 4.87]; *p* < .001).

### Correlation of different measuring methods

A significant positive correlation was found between STAI-state and VASA at all 3 time points (admission: *r* = .76, *p* < .001; skin closure: *r* = .60, *p* < .001; 2 h post caesarean: *r* = .65, *p* < .001). No significant correlations were found between cortisol and the subjective measures at any time point.

### Analysis by trait anxiety group

For a more detailed analysis we divided the patients by STAI-trait median into a high anxiety trait group (*N* = 20) and low anxiety trait group (*N* = 20). Three repeated measures ANOVAs with *time point* as the within-subject variable and *STAI-trait group* as the between subject variable were performed for the dependent variables STAI-State, VASA and cortisol respectively. A significant main effect of *STAI-trait group* was found for the VASA (*F*(1, 32) = 9.27, *p* = .005, *ηp*^*2*^ = .225), no significant main effect for STAI-state (*F*(1, 37) = 3.36, *p* = .075, *ηp*^*2*^ = .083) and no significant main effect for cortisol (*F*(1, 22) = 2.31, *p* = .143, *ηp*^*2*^ = .095). Furthermore significant *time point x STAI-trait group* interactions were revealed for STAI-state (*F*(2, 74) = 6.78, *p* = .002, *ηp*^*2*^ = .155) and VASA (*F*(2, 64) = 4.71, *p* = .012, *ηp*^*2*^ = .128) and no significant interaction was shown for cortisol (*F*(2, 44) = 2.89, *p* = .066, *ηp*^*2*^ = .116).

Planned post-hoc independent sample t-tests showed significant differences between the STAI-trait groups for STAI-state at admission (*t* (37) = 3.58, *p* = .001), VASA at admission (*t* (32) = 3.20, *p* = .002) and cortisol at skin closure (*t* (22) = 1.89, *p* = .037), indicating that women with high anxiety trait scores show significantly increased anxiety levels at the peak of the anxiety measures.

**Fig. 2:**
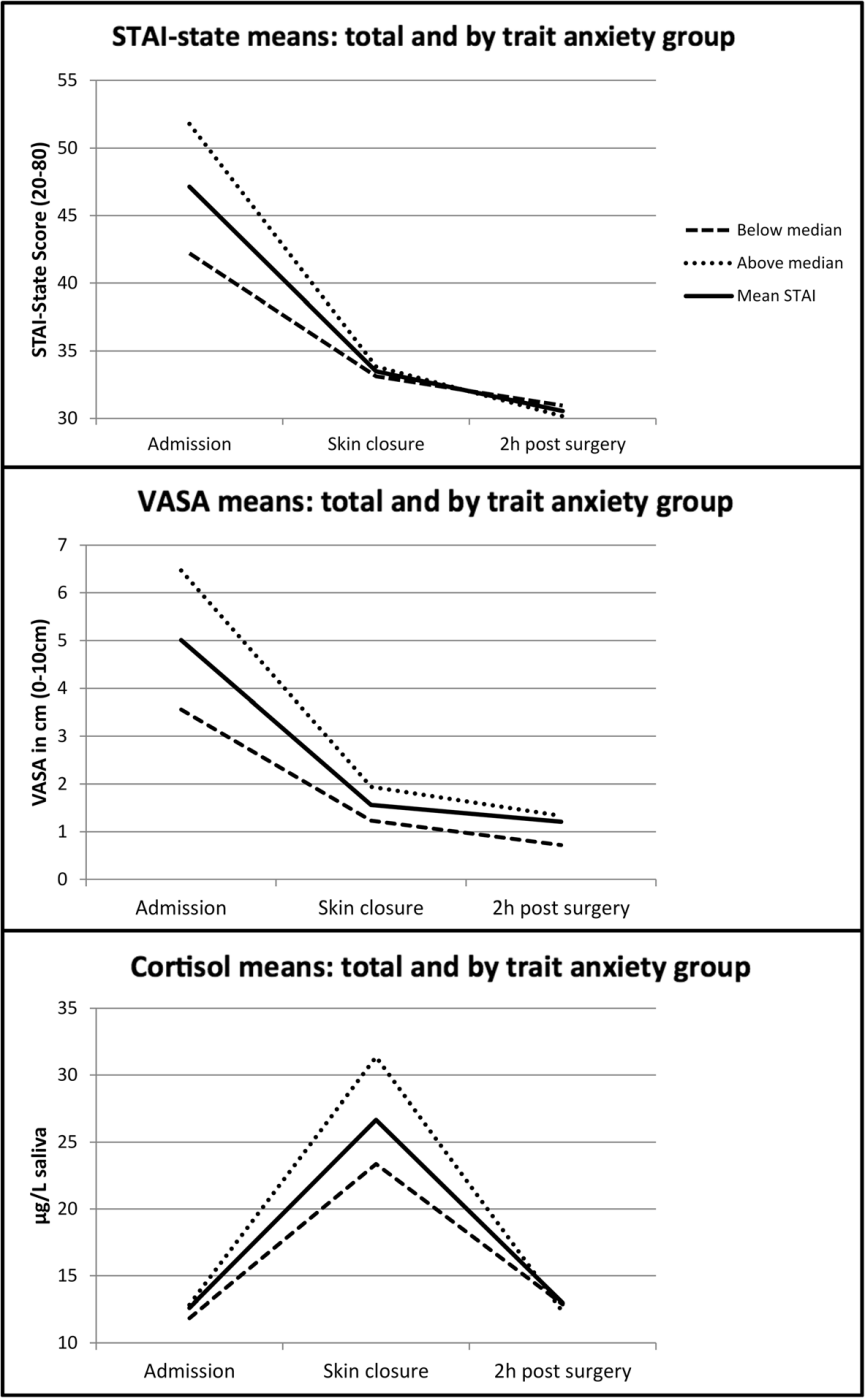
Anxiety during caesarean section. The course of the mean anxiety levels measured by STAI-state, VASA and saliva cortisol for the whole sample as well as split by Trait-group (above/below the median)

Figure 2 demonstrates the course of STAI-state, VASA and cortisol in total and separated by STAI-trait median.

## Discussion

The aim of the study was to explore level, course and extent of anxiety in women giving birth by CS by means of subjective and objective measures.

Firstly, the results highlight that subjective anxiety levels were highest before the CS. This finding is in line with studies showing preoperative anxiety in woman expecting a CS [5]. Additionally, the present study examined the course of anxiety levels on the day of the caesarean and revealed that anxiety levels evaluated by subjective measures significantly decrease from admission to skin suture. No further reduction is traceable from skin closure to two hours after the caesarean. This is an important finding as it highlights that the negative psychological impact of CS is present before the surgery and that the most significant decrease of anxiety happens during surgery in a relief like reaction reflected by STAI-state values at the time of skin closure significantly below mean STAI-state values of a normal population. This effect is further consolidated for at least the following two hours where the STAI-state values are still significantly lower in both STAI-trait groups than in a normal population. The interesting finding of anxiety scores being below the mean of the normative data is a new finding which to our knowledge has not been reported in the literature to date. However, this finding is in accordance with a study showing that the feeling of anxiety and fear was predominant before an emergency caesarean section whereas happiness overtook the mothers’ emotional state as soon as they saw their newborn baby [22].

Secondly, STAI-state and VASA highly correlated at all time-points suggesting high convergent validity between the measures as well as indicating that both measure subjective anxiety levels during CS to the same degree. This finding proposes that it would be adequate to evaluate subjective anxiety at repeated time points using the VASA in surgical settings which would be time-saving and much more practicable. A study by Davey et al., 2007 measuring anxiety in women awaiting a breast screening could also show a strong correlation between the STAI-state and the VASA suggesting that the VASA could be an adequate and more feasible approach to measure current anxiety [23]. Our results strengthen and extend this finding as we show a strong and robust correlation between these two variables at three different time points during the course of anxiety of a surgical procedure.

Furthermore, the results of the objective measure of saliva cortisol showed peak anxiety at skin suture with a significant increase of anxiety from admission to skin suture and a significant decrease from skin suture to 2 h post CS. This different pattern of anxiety development can be explained by the fact that the cortisol level in saliva samples occurs with a latency of around 30 min after the stressful event [16]. Based on this finding, the cortisol level taken at skin suture may reflect anxiety experienced during the caesarean in which tension of the expectant mother is extremely high. It is worth noting in this respect that it would be desirable for a follow-up study to include another time of measurement immediately before the CS when the woman is already in the operating room evaluating STAI-state and VASA in order to evaluate whether the subjective measures of anxiety reach a further peak at the start of the surgery. Nevertheless, a strength of the study is that three assessment points were included in order to be able to examine the course of anxiety during the day of the CS. Previous studies looking at anxiety in the context of CS measured preoperative anxiety only [4, 5, 24]. These studies are in line with our results showing high anxiety before the caesarean but the present study extends previous findings by showing that women experience a significant anxiety reduction from admission to skin suture. Furthermore, there is one study which used a qualitative approach (i.e. semistructured interviews) as well as the STAI-state questionnaire to evaluate women’s expectations and subjective experience of a planned CS which also showed that STAI-state scores (i.e. anxiety) were high before the CS and moderate after the CS which fits well to our results [25].

Additionally, the study depicts that patients with high general anxiety trait scores experience the CS with higher anxiety levels in terms of preoperative STAI-state and VASA values and intraoperative cortisol levels. This suggests that patients with higher general tendencies towards anxiety also experience stronger negative psychological side effects during the CS. It would be desirable to identify these patients at an early stage e.g. when they receive the indication for a CS, in order to provide sufficient counteractions to develop high anxiety towards the CS. As it has been shown that preoperative anxiety correlates positively to postoperative pain [7, 26, 27], the aim should be the reduction of anxiety levels before and during the CS in order to further improve wellbeing of the mothers after the CS.

In this respect, it is worth highlighting that there are interventions before or during the CS that could have a soothing impact on the patients’ anxiety levels. Several studies have shown that music before or during various surgical operations can soothe anxiety and pain levels [11, 12, 28, 29]. Referring to the CS, a study showed that music played preoperatively to the CS led to reduced self-reported anxiety and pain levels [30]. Furthermore, acupuncture could be beneficial in order to reduce anxiety levels in women waiting for a CS [31]. Another possible approach is the application of perioperative hypnosis which has been found to effectively reduce anxiety during and after CS [32].

To the best of our knowledge, this study is the first approach investigating the course of anxiety in woman having a CS looking at three assessment points on the day of the surgery and including subjective and objective measurements of anxiety. We believe that the findings of the present study are valuable for clinical implementations as the study highlights that pregnant woman awaiting a CS are extremely anxious before the operation, especially if they have a high general anxiety trait. This emphasizes that intensified attention should be provided to the pregnant women before the CS by the medical staff in order to identify patients who are prone to develop high anxiety levels and therefore requiring appropriate support or interventions.

Nevertheless, a couple of limitations warrant a comment here, which should be considered in future research. It would be desirable to conduct a study comparing the course of anxiety in woman giving birth by different modes (e.g. vaginal birth, delivery with instrumental intervention, CS) in order to examine whether different modes provoke a different development and degree of perceived anxiety. Furthermore, we acknowledge that the sample size in the present study is relatively small but comparable to other studies in the field [12, 28, 30] and according to the prospectively conducted power analysis.

## Conclusion

The present study revealed the course of anxiety during a CS by means of subjective (STAI and VASA) measures as well as saliva cortisol. The results highlight that interventions in order to reduce anxiety levels should be applied before or during the surgery and that women with high trait anxiety might benefit more from these interventions.

## Ethics approval and consent to participate

The ethics committee of the Heinrich-Heine-University Düsseldorf, Germany, has approved this study (reference number 3625). Written consent has been obtained from all patients included in the study.

## Consent for publication

Not applicable.

## Availability of data and materials

Anonymous data are available by asking the corresponding author.

## Abbreviations

ANOVA: analysis of variance
CS: caesarean section
SD: standard deviation
STAI: State-Trait Anxiety Inventory
VASA: visual analogue scale for anxiety
